# TRPM5 activation depends on a synergistic effect of calcium and PKC phosphorylation

**DOI:** 10.1038/s42003-024-06054-3

**Published:** 2024-03-27

**Authors:** Alaa Nmarneh, Avi Priel

**Affiliations:** The Institute for Drug Research, School of Pharmacy, Faculty of Medicine, The Hebrew University of Jerusalem, Ein Karem, Jerusalem, 9112102 Israel

**Keywords:** Transient receptor potential channels, Kinases

## Abstract

Transient receptor potential melastatin 5 (TRPM5) is a calcium-activated monovalent-specific ion channel involved in insulin secretion and taste transduction, making it an attractive target for drug development in various pathologies. While TRPM5 activation involves ligand binding to Gq/G-protein coupled receptors (GPCR) and subsequent elevation of intracellular calcium levels, recent reports suggest the need for additional molecular determinants. Hence, the mechanism of TRPM5 activation remains to be elucidated. Here, we show that PKC phosphorylation and the elevation of intracellular Ca^2+^ levels are required for TRPM5 activation, with PKC phosphorylation being crucial for channel-evoked currents, primarily at physiological membrane potentials. In contrast, physiological relevant calcium levels alone only induce TRPM5 activation at positive voltages. Our findings highlight the necessity of coordinated intracellular calcium release and PKC phosphorylation for TRPM5 activation. Thus, our results suggest that regulation of PKC activity could be a promising therapeutic target for diseases associated with TRPM5 modulation.

## Abstract (web summary)

Both PKC phosphorylation and elevated calcium levels are required for TRPM5 activation, the phosphorylation is crucial for channel-evoked currents at physiological membrane potentials.

## Introduction

TRPM5, also known as transient receptor potential cation channel subfamily M (melastatin) member 5, is a voltage-modulated monovalent cation-selective ion channel protein activated directly by increasing intracellular calcium ions, but is impermeable to it^1,2^. TRPM5 is abundant in pancreatic beta cells and is responsible for the production and release of insulin^3,4^. In addition, TRPM5 is highly expressed in taste buds and is involved in the transduction of sweet, bitter, and umami taste signals^5^. Thus, TRPM5 plays numerous crucial roles in various physiological processes, and is a promising therapeutic target. However, the development of drugs that affect intracellular calcium-activated monovalent cation channels (such as TRPM4 and TRPM5) poses a significant challenge^6^. Thus, defining molecular targets that modulate TRPM5 activity may serve as potential drugs for pathophysiological conditions such as diabetes and other gastrointestinal conditions.

As previously described, the activation mechanism of TRPM5 involves a multistep process^7^. Upon ligand binding (i.e., tastant molecule, glucose stimulation) to the Gαq G protein-coupled receptor (Gq/GPCR)^8^, a cascade of intracellular signaling pathways is initiated, leading to the breakdown of phosphatidylinositol bisphosphate (PIP2) into diacylglycerol (DAG) and inositol trisphosphate (IP3), IP3 binds to its receptors in the endoplasmic reticulum and cause calcium release from the stores^3,5^. An increase in cytosolic Ca^2+^ concentration activates TRPM5 channels, allowing the entry of sodium ions (Na^+^) into the cell. This ion influx depolarizes the cell membrane, generating action potentials that propagate TRPM5 signals^9^. In summary, the activation of TRPM5 is a complex process that involves the integration of ligand binding, intracellular calcium-dependent signaling, and ion channel opening^10^. Previous studies have suggested that TRPM5 is activated via a store-operated mechanism^11,12^. Several studies have highlighted the possible cellular mechanisms of IP3-mediated Ca^2+^ release and PIP2-enhancement sensitivity to Ca^2+,^^2,13–15^. However, others have suggested that TRPM5 activation requires GPCR activation, but is calcium-independent^16^. Therefore, the precise molecular signaling mechanisms that govern TRPM5 modulation or activation are yet to be fully elucidated.

Hence, we hypothesized that TRPM5 activation requires multiple components of the Gq/GPCR cascade. Our findings shed light on the pivotal role of protein kinase C (PKC) phosphorylation in Gq/GPCR-mediated activation of TRPM5, departing from the conventional Ca^2+^-centric view^17^. We showed that PKC phosphorylation and the elevation of intracellular Ca^2+^ levels are required for TRPM5 activation. Moreover, our findings suggest that PKC phosphorylation is essential for channel-evoked currents, mainly at relevant physiological membrane potentials (i.e., negative voltages). In contrast, calcium alone could induce TRPM5 activation solely at positive voltages. Overall, in this study, we describe sequential molecular events starting from Gq/GPCR activation through PKC phosphorylation of a single amino acid, leading to the subsequent activation of TRPM5. Hence, our results suggest that regulation of PKC activity may serve as a potential drug target to modulate TRPM5 activity under pathophysiological conditions.

## Materials and methods

### Molecular biology

Mouse TRPM5 (*mTrpm5*) was a gift from Craig Montell (Addgene plasmid # 85189), Gq designer receptors exclusively activated by designer drugs; Gq/Dreadd (Addgene plasmid # 45547), Gs/Dreadd (Addgene plasmid # 45549), and Gi/Dreadd (Addgene plasmid # 45548); a gift from Bryan Roth, University of North Carolina, Chapel Hill, NC, USA, and Human histamine receptor H1 (hH1R) genes were subcloned into pCDNA3.1 (+). The fusion EGFP-TRPM5 construct was designed using standard PCR techniques as follows: the TRPM5 coding sequence was subcloned into EGFP to generate an N-terminal fusion-enhanced EGFP-TRPM5 protein inserted into the pCDNA3.1+ vector to confirm successful transfection. Human Gαq, GαqQ209L, and Gαi2 were purchased from the cDNA Resource Center (www.cdna.org). Chimeras between Gαq and Gαi2 were designed using the HIFI DNA Assembly Cloning Kit (New England Biolabs). Site-directed mutagenesis was performed on mTRPM5 using the Q5 Site Mutagenesis Kit (New England Biolabs) (the oligos sequence is attached (Supplementary Table 1)). All constructs were verified using DNA sequencing (Hy-Labs, Rehovot, Israel).

### Cell culture and transient transfection

Human embryonic kidney 293 T (HEK293T) cells (ATCC, CRL-3216) were cultured in Dulbecco’s modified Eagle’s medium (DMEM) supplemented with 10% fetal bovine serum, 1% penicillin-streptomycin, and 25 mM HEPES (pH-7.3). The cells were passaged twice per week (up to 15 passages) and grown at 37 °C and 5% CO_2,_ as previously described^18^. Cell transfection was carried out as follows: Human embryonic kidney 293 T (HEK293T) cells were transfected with a total of 1 μg of DNA using Lipofectamine 3000 transfection reagent (Thermo Fisher Scientific, MA) with Opti-MEM I Reduced Serum Medium (Invitrogen, MA, USA). Transfections were performed in 12-well plates containing ~3 × 10^5^ cells 24 h before analysis. Cells were plated on 0.1 mg/ml PDL-coated glass coverslips (12 mm) and incubated at 37 °C (5% CO_2_) for at least 2 h before electrophysiological analysis.

### Electrophysiology

Whole-cell patch-clamp recordings from transfected HEK293T cells were performed by an Axopatch 200B patch-clamp amplifier (Molecular Devices, CA). Membrane currents were digitized using a Digidata 1440 A interface board and pCLAMP 10.7 software (Molecular Devices, CA) with a sampling frequency set to 5 kHz and low-pass filtered at 2 kHz, as previously described^18–21^. The holding voltage was −40 mV. Patch electrodes were fabricated from borosilicate glass using the P1000 Micropipette Puller (Sutter Instrument, CA, and USA) and fire-polished using the microforge MF-900 (Narishige, Tokyo, Japan) to the resistance of 2–4 MΩ. The standard pipette solution (3 μM free Ca^2+^) comprised 1.91 mM CaCl_2_, 2 mM EGTA, 140 mM CsCl, and 10 mM HEPES, adjusted to pH 7.2 with CsOH. For nominally free recordings, the pipette solution contained 120 mM CsCl, 10 mM NaCl, 1 mM MgCl_2_, and 10 mM HEPES adjusted to pH 7.4, with CsOH. Solutions containing different free Ca^2+^ concentrations were prepared by adding an appropriate amount of CaCl_2_ to stock solutions of EGTA (2 mM), CsCl (140 mM), and HEPES (10 mM). All Ca^2+^ concentrations were calculated using the MAXCHELATOR software (Chris Patton, Stanford University, CA, USA). The bath solution contained:140 mM NaCl, 2.5 mM KCl, 1 mM MgSO_4_, 1.8 mM CaCl_2_, and 10 mM HEPES and was adjusted to pH 7.4 by NaOH. After establishing the whole-cell configuration, ramps (1 s^−1^) were administered from −100 mV to + 100 mV at 2 s intervals, and cells were perfused using the ValveBank perfusion system (AutoMate Scientific, CA, USA). All the experiments were performed at room temperature.

### Chemicals

All salts and buffers were purchased from Sigma-Aldrich (St. Louis, MO, USA). U73122 and BIM I (GF 109203X) were purchased from Tocris Biosciences (Bristol, UK). Clozapine *N*-oxide (CNO) and histamine dihydrochloride were purchased from Sigma-Aldrich (St. Louis, MO, USA). All drugs were dissolved in DMSO according to the manufacturer’s protocol.

### Statistics and reproducibility

We assumed a normal distribution for our data but did not formally check it. The sample size (*n*) indicates the number of individual cells. Transfected cells from at least three different transfections per construct were used to account for potential day-to-day variation. Results are presented as mean ± SEM if not stated otherwise, but individual data points are shown in the figures, allowing for visual assessment of data variation.

*P* values were calculated using paired *t* tests for within-cell comparisons (e.g., before and after drug or treatment application) and unpaired *t* tests for between-cell comparisons involving two groups. For analyses concerning multiple groups, one or two-way ANOVA was used according to the number of independent variables or factors being analyzed, followed by Tukey or Dunnett’s post hoc tests as appropriate.

All statistical data were analyzed using Prism 10 software (GraphPad Software, CA, USA). Electrophysiological analysis and traces were performed using pCLAMP 10.7 software (Molecular Devices, CA, USA). Dose-response curves were fitted using the sigmoidal Hill equation as follows:

$$\frac{I}{{I}_{\max }}=\frac{{[X]}^{n}}{E{{C}_{50}}^{n}+{[X]}^{n}}$$

Where: I = measured current, I_max_ = maximal current at a saturating dose (pre-measured for each construct), X = tested agonist concentration, EC_50_ = the calculated concentration that elicits 50% of maximal current, and n = Hill coefficient.

### Reporting summary

Further information on research design is available in the Nature Portfolio Reporting Summary linked to this article.

## Results

### Activation of the Gq/GPCR pathway positively modulates the TRPM5 currents

**Fig. 1:**
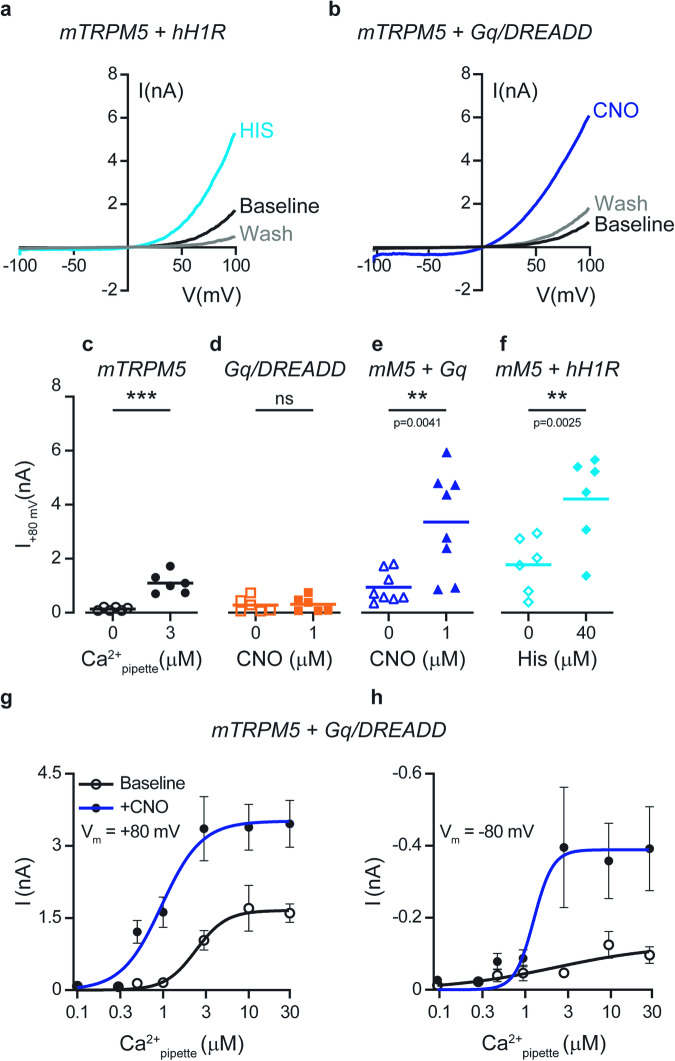
Activation of the Gq/GPCR pathway positively modulates Ca^2+^-evoked TRPM5 currents by increasing calcium efficacy and potency. Representative current-voltage relationship traces in HEK293T cells transiently co-expressing the mTRPM5 with hH1R (**a**) or Gq/DREADD (**b**). Currents were recorded with 3 μM Ca^2+^ in the recording pipette before (Baseline; black trace) and after (Wash; gray trace) the GPCR agonizts application histamine (HIS; 40 μM; cyan line; left) or CNO (CNO; 1 μM; blue line; right). Currents were recorded using whole-cell patch-clamp recording (in 1 s^−1^ voltage ramps between −100 and +100 mV) *n* = 6−10. Note that both Gq/GPCR agonizts dramatically increase the mTRPM5 current. **c** Mean/Scatter dot plot representing the current mean amplitude in +80 mV of HEK293T cells transiently expressing mTRPM5 with 3 μM Ca^2+^ in the recording pipette (full circles) or without calcium (empty circles). Statistical significance was determined by unpaired t-test when ***p ≤ 0.001. **d** Mean/Scatter dot plot representing the current mean amplitude in +80 mV of HEK293T cells transiently expressing Gq/DREADD with 3 μM Ca^2+^ in the recording pipette before (empty squares) and after (full squares) CNO (1 μM) application. Statistical significance was determined by paired *t* test when ns, not statistically significant. Mean/Scatter dot plot representing the current mean amplitude in +80 mV of HEK293T cells transiently co-expressing the mTRPM5 with Gq/DREADD (**e**) or hH1R (**f**) with 3 μM Ca^2+^ in the recording pipette before empty triangles (**e**) or empty rhombuses (**f**) and after Gq/GPCR agonist application (CNO (CNO; 1 μM), full triangles (**e**) or histamine (40 μM), full rhombuses (**f**)). Statistical significance was determined by paired *t* test. Concentration-response relationships for intracellular (i.e., pipette) calcium of HEK293T cells co-expressing mTRPM5 and Gq/DREADD before (empty circles) and after (full circles) the application of CNO (1 μM) at +80 mV (**g**) or −80 mV (**h**). Each point represents the average (±SEM) response of 6−9 cells. Solid lines are fit to the Hill equation Baseline (+80 mV; black line), EC_50_ = 2.4 ± 0.4 μM with *n* = 2.4 ± 1.0, after CNO (+80 mV; blue line): EC_50_ = 0.9 ± 0.2 μM with *n* = 1.8 ± 0.7. Baseline (−80 mV; black line), EC_50_ = 2.4 ± 4.3 μM with *n* = 0.7 ± 0.5, after CNO (−80 mV; blue line): EC_50_ = 1.3 ± 0.6 μM with *n* = 3.9 ± 5.9.

TRPM5 has been suggested to be mainly activated by calcium release from its intracellular stores downstream of the Gq/GPCR signaling cascade. Other members of the TRPM family have been shown to be regulated directly by other components of the Gq/GPCR pathway^10,22,23^. However, it remains to be demonstrated whether different components of this pathway regulate or modulate TRPM5 channels. To this end, we tested evoked TRPM5 currents under constant calcium concentrations. We first analyzed the response of mouse TRPM5 (mTRPM5) to activation of the Gq-dominant receptor, human histamine receptor 1 (hH1R) (Fig. 1a).

Using the whole-cell configuration of the patch-clamp technique, we applied a constant free calcium concentration (3 µM) through the pipette. We used this calcium concentration because it was previously shown to be near saturation^14^, thus evoking a substantial current that can be increased. The application of histamine to HEK293T cells co-expressing mTRPM5 and hH1R significantly increased the evoked current compared to that evoked by calcium alone (Fig. 1a, f). To specifically target the Gq/GPCR pathway, we used the Designer Receptor Exclusively Activated by Designer Drugs, Gq/DREADD, which was engineered to be activated solely through the synthetic exogenous chemical clozapine N-oxide (CNO)^24,25^. Exposure of HEK293T cells co-expressing mTRPM5 and Gq/DREADD to CNO resulted in similar current augmentation (compare Fig. 1a, b and Fig. 1e, f). Importantly, this Gq/GPCR-dependent TRPM5 current enhancement was observed only when both proteins were coexpressed (Fig. 1b−d and Supplementary Fig. 1a, b). Moreover, positive Gq/GPCR modulation was observed after steady-state desensitization of the TRPM5 current (Supplementary Fig. 1c, d).

Although we applied a constant calcium concentration through the pipette and reached diffusional equilibrium between the pipette and the cell (Supplementary Fig. 1), Gq-dependent current amplification may result from increased calcium levels owing to intracellular store depletion. Thus, increasing the calcium concentration in the pipette should saturate the TRPM5-evoked response and minimize the Gq-positive modulation. To test this hypothesis, we performed a dose-response analysis with and without Gq/GPCR activation. We observed a significant current increase at all calcium-activating concentrations (Fig. 1g, h). Moreover, a shift in the potency and efficacy of the Ca^2+^-evoked TRPM5 currents was observed at both positive and negative voltages (Fig. 1g, h). Importantly, at negative potentials, the calcium-evoked TRPM5 current was minuscule, without activation of the Gq/GPCR pathway. Previous studies have shown that high non-physiological relevant calcium concentrations increase the inward current of TRPM5^10,26^. Therefore, we tested whether increasing the intracellular calcium concentrations may affect the Gq/GPCR-dependent TRPM5 current enhancement. Indeed, elevating the intracellular calcium concentrations evokes higher TRPM5 baseline currents at both positive and negative potentials (Supplementary Fig. 2a−c). Notably, the Gq/GPCR activation still enhances the TRPM5 calcium-evoked currents even at high non-physiological calcium levels (Supplementary Fig. 2). Hence, our results indicate that TRPM5 activation downstream of the Gq pathway involves yet-to-be-defined positive modulation that dramatically augments the calcium-evoked current at both negative (i.e., physiologically relevant voltages) and positive potentials.

### PKC activation is required for the observed positive modulation of TRPM5

**Fig. 2:**
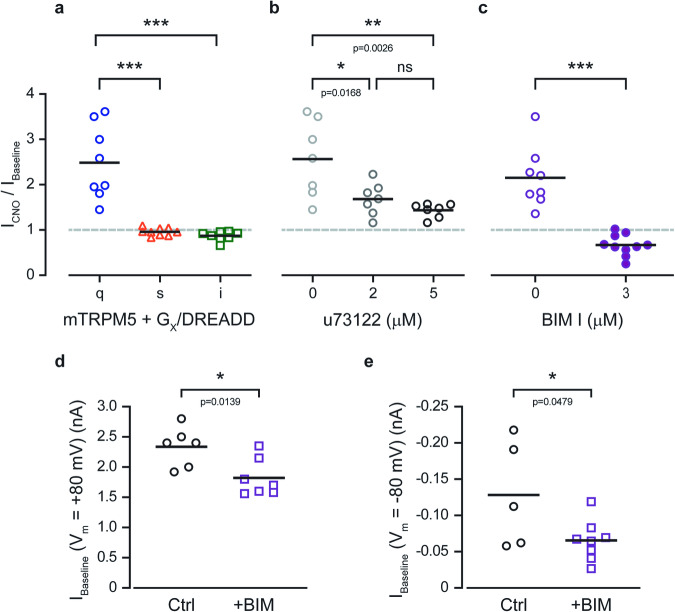
PKC activation governs the positive modulation of TRPM5 by the Gq pathway. **a** Mean/Scatter dot plot representing the CNO (1 μM)-current amplification at +80 mV with 3 μM Ca^2+^ in the recording pipette of HEK293T cells transiently co-expressing the mTRPM5 with Gq/DREADD (q; blue circles) or Gs/DREADD (s; orange triangles) or Gi/DREADD (i; green squares) (*n* = 6−10). Statistical significance was determined using ANOVA multi-comparison test when ***p ≤ 0.001. **b** Mean/Scatter dot plot representing the CNO (1 μM)-current amplification at +80 mV with 3 μM Ca^2+^ in the recording pipette of HEK293T cells transiently co-expressing the mTRPM5 with Gq/DREADD in the presence of different concentrations of the PLC inhibitor U73122 (*n* = 7). Statistical significance was determined using ANOVA multi-comparison test when ns, not statistically significant. **c** Mean/Scatter dot plot representing the CNO (1 μM)-current amplification at +80 mV with 3 μM Ca^2+^ in the recording pipette of HEK293T cells transiently co-expressing the mTRPM5 with Gq/DREADD with and without the PKC inhibitor BIM I (*n* = 8−10). Statistical significance was determined using the unpaired *t* test when ***p ≤ 0.001. Mean/Scatter dot plot representing the baseline current of 3 μM Ca^2+^ in the recording pipette at +80 mV (**d**) and −80 mV (**e**) of HEK293T cells transiently co-expressing the mTRPM5 with Gq/DREADD with (+BIM) and without (Ctrl) the PKC inhibitor BIM I (*n* = 5−8). Statistical significance was determined using the unpaired *t* test.

To define which of the Gq pathway components are required for TRPM5 positive modulation, we first verified that this phenomenon is unique to the Gq/GPCR cascade. We co-expressed mTRPM5 with Gq/, Gs/, or Gi/DREADD receptors and recorded Ca^2+^-dependent currents before and after CNO application. We observed a significant current modulation only when Gq/DREADD was activated (Fig. 2a).

Upon ligand binding to GPCRs, the Gα and Gβγ subunit complexes can independently propagate a cascade of intracellular signaling events^27,28^. Therefore, we aimed to determine whether the Gαq subunit affects TRPM5 directly, as has been previously shown for other TRPM members (e.g., TRPM3 and TRPM8)^23,27–29^. To this end, we co-expressed the TRPM5 with previously described Gαq mutants with Gq/DREADD (Supplementary Fig. 3). We overexpressed various Gαq isoforms: wild-type, constitutively active (Gαq (Q209L))^30^, dominant negative (Gαq (D277N))^31^, and the non-PLCβ binding mutant (3Gαqiq (Q209L))^32^. Our results show that co-expression of the WT Gα_q_ subunit reduced the baseline current (Supplementary Fig. 3a) and eliminated the TRPM5 currents amplification by the Gq/DREADD cascade (Supplementary Fig. 3b). A possible explanation of those results is that the overexpression of the Gα_q_ subunit inhibits both the leak response of the endogenous G proteins and the GPCR response due to the lack of sufficient amounts of GTP (required for the detachment of the G-proteins from the receptor). To test this possibility, we added GTP (1 mM) to the pipette solution, which rescued the phenomena to some extent (Supplementary Fig. 3) but did not recover the baseline and CNO-dependent response. Nevertheless, our results point to the necessity of the Gα_q_ subunit activation within activation of the Gq cascade for the TRPM5 modulation by the Gq/GPCR. Next, to test whether the Gα_q_ subunit modulates the TRPM5 current by binding directly to the channel, we used the chimeric Gα_q_ subunit (3Gα_qiq_). In this chimera, the PLCβ- binding region on the Gαq domain is replaced by the corresponding part of the Gαi_2_ subunit and includes the Q209L mutation. Thus, this chimera is a constitutively active Gα_q_ subunit that cannot activate the Gq pathway. Co-expression of cells with mTRPM5, Gq/DREADD, and 3Gα_qiq_ resulted in a similar response, both the baseline current (Supplementary Fig. 3a) and CNO-dependent response (Supplementary Fig. 3b), as the control. These results indicate that the Gα_q_ subunit cannot bind directly to TRPM5. Moreover, the dominant-negative mutants of Gα subunits have been extensively used to delineate G‐protein signaling pathways. Therefore, we generated two dominants negative Gα_q_ subunits. The first mutant, termed Gα_q_(D277N), which strength the Gα-Gβγ protein interface and reduces the response caused by the wild‐type Gα_q_, and the second mutant, termed Gα_q_(Q209L/D277N), binds to the activated Gβγ resulted in a block of the Gq pathway. While co-expression of cells with mTRPM5, Gq/DREADD, and Gα_q_(D277N) resulted in baseline current reduction (Supplementary Fig. 3a) due to its more durable binding to the Gβγ, application of CNO resulted in standard TRPM5 current modulation as expected for these weak mutant dominant-negative properties (Supplementary Fig. 3b). However, co-expression of mTRPM5, Gq/DREADD, and Gα_q_ (Q209L/D277N) resulted in a significant decrease of the baseline current (Supplementary Fig. 3a) and no recorded effect of CNO (Supplementary Fig. 3b). Thus, our results indicate that, unlike other TRPM channels, the Gα_q_ subunit cannot produce TRPM5 modulation independently of the Gq signaling cascade. Thus, phospholipase C (PLC) inhibition should abolish Gq-dependent TRPM5 current amplification. Indeed, PLC inhibition by the broad-spectrum inhibitor U73122^25^ significantly diminished the CNO-dependent modulation of TRPM5 in a dose-dependent manner (Fig. 2b). We applied U73122 using a pipette to avoid toxicity when applied extracellularly.

Recent studies have demonstrated that TRPM channels, such as the cold receptors TRPM8^22^ and TRPM4^33^, are regulated by PKC phosphorylation downstream of the Gq/GPCR. To test this, we inhibited PKC-dependent processes using the potent PKC inhibitor GF109203X (BIM I). Pre-incubation of cells co-expressing mTRPM5 and the Gq/DREADD receptor with BIM I (3 µM) abolished Gq-dependent modulation of the channel current (Fig. 2c). Moreover, the baseline current (3 µM Ca^2+^ in the recording pipette) was significantly decreased at both negative and positive potentials in the presence of BIM I, probably because of the inhibition of PKC basal phosphorylation (Fig. 2d, e). Thus, our results indicated that the Gq/GPCR pathway modulates the TRPM5 channel by activating PKC.

### Phosphorylation of Serine 129 mediates the Gq-dependent modulation

Our results suggested that PKC is pivotal for Gq/GPCR TRPM5 modulation (Fig. 2c). However, this enzyme activity may be either direct (i.e., phosphorylation of TRPM5) or indirect (e.g., phosphorylation of signaling-participating protein(s)).

To examine whether direct phosphorylation of TRPM5 by PKC is required for the current augmentation, we used different kinase site predicting software: NetPhos 3.1(NetPhos3.1)^34^ and PPSP (Prediction of PK-Specific Phosphorylation Site)^35^. We focused on sites predicted to be intracellular or on the membrane cytosol interface, according to recently published cryo-EM structures of TRPM5^26^ and TRPM4^36–40^. Fifteen predicted sites with the highest scores were identified and analyzed (Table 1).

**Table 1:** Predicted phosphorylation sites of PKC on TRPM5

mTRPM5 construct #X	Netphos Scores > 7.0	Prediction of PK-Specific Phosphorylation Site PPSP	I_CNO_/ I_Baseline_ ( + 80 mV)	I_CNO_/ I_Baseline_ (−80 mV)	Number of replicates (*n*)
WT			4.2 ± 0.7**	10.3 ± 3.76*	10
33 S	+	+	1.8 ± 0.25*	4.5 ± 1.71*	6
98 S	+	-	2.4 ± 0.32*	4.6 ± 1.58	6
127 S	+	-	2.6 ± 0.37	14.1 ± 8.6	5
129 S	unsp^a^	+	1.0 ± 0.36	1 ± 0.05	5
130 T	+	-	1.9 ± 0.22*	2.3 ± 0.48*	7
534 T	+	-	2.2 ± 0.66	2.0 ± 0.36*	5
569 T	unsp^a^	-	1.2 ± 0.16	1.6 ± 0.44	6
633 T	+	-	1.4 ± 0.12*	3.4 ± 1.74	5
721 T	+	-	2.2 ± 0.37**	9.7 ± 5.12	7
822 T	+	-	3.5 ± 1.8*	7.5 ± 3.0*	6
845 T	+	-	2.6 ± 0.17**	2.2 ± 0.42	6
1055 T	+	-	2.6 ± 0.54**	4.4 ± 1.07	5
1064 T	+	-	2.1 ± 0.32*	2.2 ± 0.46*	5
1106 S	+	-	1.8 ± 0.25*	2.3 ± 0.4	5
1142 S	+	+	3.6 ± 0.52**	3.9 ± 1.35*	6

Phosphorylation site prediction was performed using Netphos and PPSP software; sites with scores higher than 7/10 were marked with (+) and lower with (–), respectively. The CNO fold-increase in current was calculated as the average ±SEM for (*n* = 5−10) cells for each site mutation (TRPM5 (#X)) at ±80 mV.

Note that threonine at site 130 was converted to valine rather than alanine because of the low expression of the alanine substitution construct. Statistical significance was determined using a paired *t* test for each construct when **p ≤ 0.01, *p ≤ 0.05.

^a^unsp - active kinase site for non-specific prediction.

**Fig. 3:**
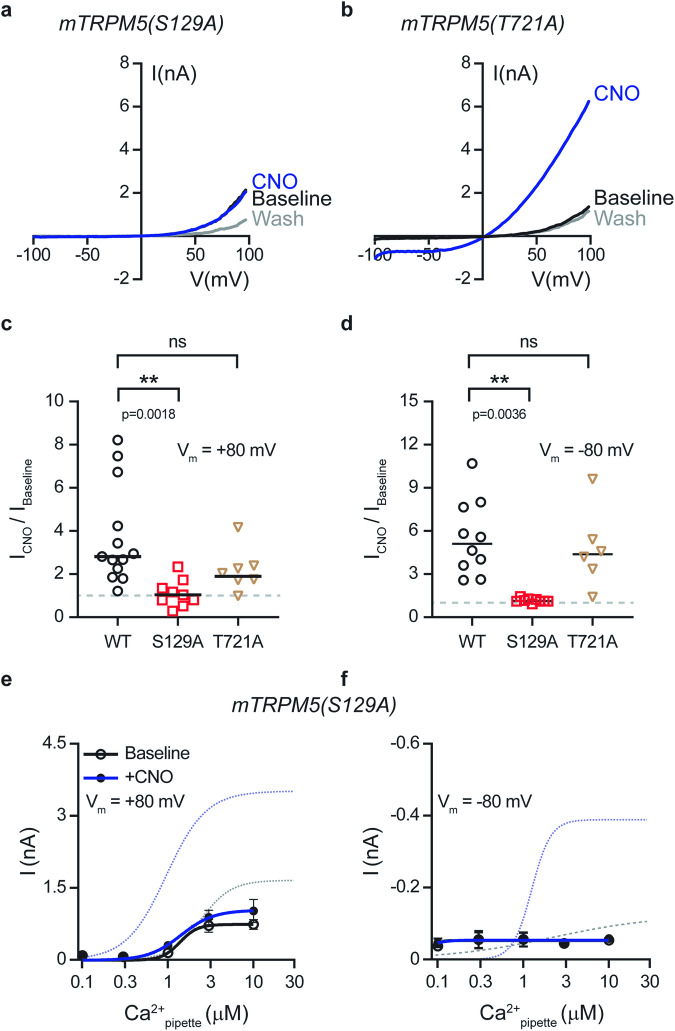
Point mutation at mTRPM5 (S129) abolishes the Gq/GPCR modulation of TRPM5. Representative current-voltage relationship traces in HEK293T cells transiently co-expressing the mutated mTRPM5 constructs (S129A (**a**) or T721A (**b**)) with Gq/DREADD. Currents were recorded with 3 μM Ca^2+^ in the recording pipette before (Baseline; black trace) and after (Wash; gray trace) the CNO (CNO; 1 μM; blue line) application. Currents were recorded using whole-cell patch-clamp recording (in 1 s − 1 voltage ramps between −100 and +100 mV) (*n* = 6−11). Mean/Scatter dot plot representing the CNO (1 μM)-current amplification at +80 mV (**c**) and −80 mV (**d**) with 3 μM Ca^2+^ in the recording pipette of HEK293T cells transiently co-expressing mTRPM5 (WT; black circles) or the mutated mTRPM5 constructs (S129A (magenta squares) or T721A (brown triangles)) with Gq/DREADD (*n* = 6−13). Statistical significance was determined using ANOVA multi-comparison test when ns, not statistically significant. Concentration-response relationships for intracellular calcium (i.e., pipette) of HEK293T cells co-expressing mTRPM5(S129A) and Gq/DREADD before (Baseline; empty circles) and after the application of CNO (1 μM) (+CNO; full circles) at +80 mV (**e**) and −80 mV (**f**). Each of the obtained set data points was fitted with the Hill equation (black line and blue line, Baseline and CNO application, respectively). Baseline (+80 mV; black line): EC_50_ = 1.3 ± 0.5 μM with *n* = 4.2 ± 4.2, while after CNO (+80 mV; blue line) EC_50_ = 1.4 ± 0.5 μM with *n* = 2.2 ± 1.6. Baseline (−80 mV; black line): EC_50_ = 0.09 μM with unstable n, while after CNO (−80 mV; blue line): EC_50_ = 0.06 ± 1.1 μM with *n* = 4.0 ± 154.6. Values are means ± SEM for 6−9 cells at each point. Dashed lines represent the WT mTRPM5 dose response as presented in Fig. 1g, h.

To determine the site necessary for Gq/GPCR TRPM5 modulation, we performed an alanine mutagenesis scan of each predicted site, followed by a patch-clamp recording of the Gq/GPCR modulation of TRPM5 currents. Converting serine to alanine at position 129 (mTRPM5 (S129A)) abolished the Gq-dependent modulation (Fig. 3a, c, d). Although other predicted sites demonstrated a shift in the CNO-fold current increase (Table 1 and Fig. 3), S129 was the only site predicted by both software programs and eliminated Gq/GPCR modulation (compare S129 to S33 and S1142).

PKC phosphorylation sites were screened using a fixed concentration of calcium ions (3 µM) in the intracellular milieu. It is possible that these mutations reduced CNO-dependent TRPM5 currents by shifting TRPM5 calcium sensitivity. To examine this possibility, we performed a dose-response analysis of the TRPM5 (S129A) mutant with and without Gq/GPCR activation. Non-significant current augmentation due to Gq activation was observed at all calcium concentrations (Fig. 3e, f). Furthermore, neutralization of the predicted phosphorylation site (S129A) significantly minimized the shift in potency and efficacy of the Ca^2+^-evoked TRPM5 currents, both at positive and negative voltages (compare Fig. 3e, f and Fig. 1g, h). Thus, our results suggest that the phosphorylation of S129 by PKC is pivotal for Gq/GPCR modulation.

### TRPM5(S129D) has similar characteristics as the PKC-dependent phosphorylated channel

**Fig. 4:**
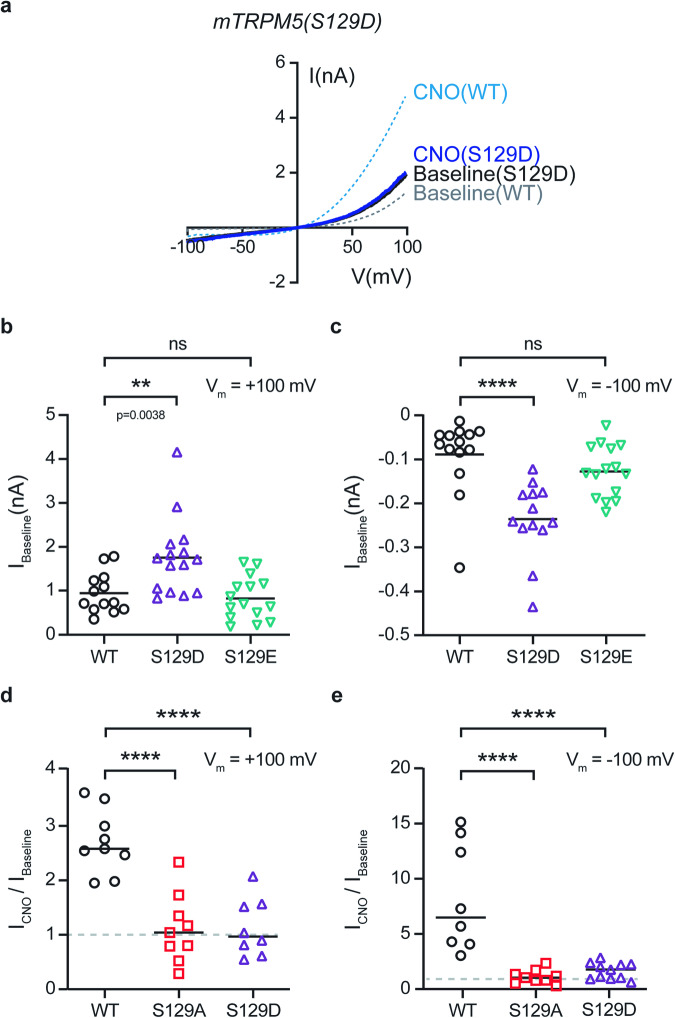
The phospho-mimic mutation S129D demonstrates similar characteristics to the Gq/GPCR-dependent TRPM5 phosphorylation. **a** Representative current-voltage relationship traces in HEK293T cells transiently co-expressing the mutated mTRPM5 (S129D) with Gq/DREADD. Currents were recorded with 3 μM Ca^2+^ in the recording pipette before (Baseline; black trace) and following CNO application (CNO; 1 μM; dark blue line). Currents were recorded using the whole-cell configuration (1 s^−1^ voltage ramps between −100 and +100 mV) (*n* = 15). Dashed lines represent the averaged WT mTRPM5 response for 3 μM intracellular Ca^2+^ (baseline; gray trace) and for CNO application (CNO; 1 μM; light blue line) (*n* = 6−7). Mean/Scatter dot plot representing the baseline current of 3 μM Ca^2+^ in the recording pipette at +100 mV (**b**) and −100 mV (**c**) of HEK293T cells transiently co-expressing the mTRPM5 with (WT; black circles) or the mutated mTRPM5 constructs (S129D (magenta triangles) or S129E (mint triangles)) (*n* = 13−15). Statistical significance was determined using the ANOVA multi-comparison test when****p ≤ 0.0001, and ns, not statistically significant. Note that S129D demonstrates a significantly higher baseline current at both voltages. Mean/Scatter dot plot representing the CNO (1 μM)-current amplification at +100 mV (**d**) and −100 mV (**e**) with 3 μM Ca^2+^ in the recording pipette of HEK293T cells transiently co-expressing mTRPM5 (WT; black circles) or the mutated mTRPM5 constructs (S129A (red squares) or S129D (purple triangles)) with Gq/DREADD (*n* = 8−10). Statistical significance was determined using the ANOVA multi-comparison test when ****p ≤ 0.0001.

To further analyze whether the phosphorylation of residue S129 underlies the Gq/GPCR modulation of TRPM5, we generate phosphomimetic substitutions. Previous studies demonstrated that substituting putative phosphorylated residues to aspartate or glutamate may mimic the behavior of the phosphorylated form of the protein^41,42^. Of note, substitutions to these amino acids may not mimic the phosphorylated state successfully; however, it is strong evidence of residue phosphorylation that such substitutions result in similar characteristics as the phosphorylated protein. Substitution of residue S129 to aspartate (D) resulted in a significant increase of the calcium-evoked TRPM5 current (baseline) in both positive and negative voltages (Fig. 4a−c). On the other hand, substituting residue S129 to glutamate (E) did not change the calcium-evoked current of TRPM5 (Fig. 4b, c). As expected, activation of the Gq/GPCR pathway by CNO did not increase the calcium-evoked current of both S129A and S129D (Fig. 4d, e). Thus, the mutated channel TRPM5(S129D) demonstrates similar characteristics as PKC-phosphorylated TRPM5.

### TRPM5 activation required both calcium release and PKC phosphorylation

**Fig. 5:**
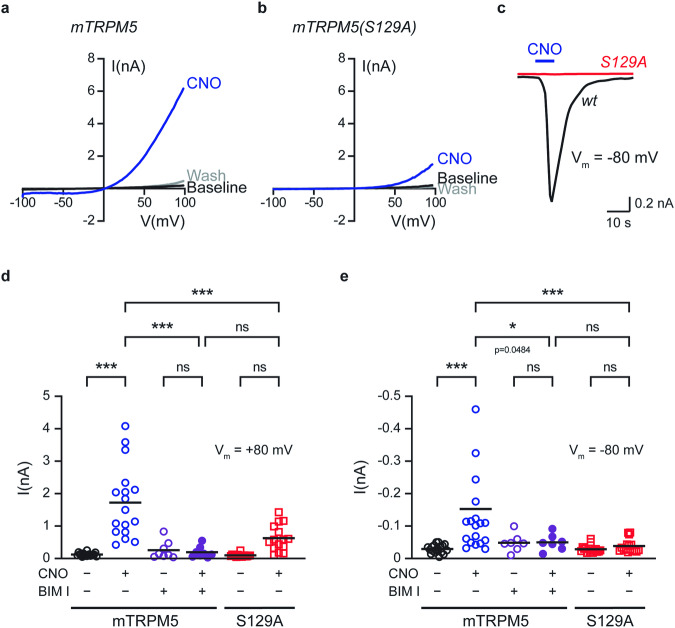
Both calcium release from intracellular stores and PKC-phosphorylation governs TRPM5 activation. Representative current-voltage relationship traces in HEK293T cells transiently co-expressing the WT mTRPM5 (**a**) or the mutated mTRPM5 (S129A) (**b**) with Gq/DREADD. Currents were recorded using a nominally free pipette solution before (Baseline; black trace) and after (Wash; gray trace) the CNO (CNO; 1 μM; blue line) application. Currents were recorded using the whole-cell configuration (1 s − 1 voltage ramps between −100 and +100 mV) (*n* = 9−15). **c** Representative whole-cell current trace (Vm = −80 mV) from HEK293T cells transiently co-expressing the WT mTRPM5 (wt, black trace) or the mutated mTRPM5 (S129A) (S129A, red trace) with Gq/DREADD in response to CNO (1 μM) application. Currents were recorded using a nominally free pipette solution (*n* = 9). Note that the S129A mutation abolishes the Gq/DREADD response at negative potentials. Mean/Scatter dot plot representing the currents of HEK293T cells transiently co-expressing the mTRPM5 or the mutated mTRPM5(S129A) with Gq/DREADD using a nominally free pipette solution at +80 mV (**d**) and −80 mV (**e**). Currents were recorded with or without applying CNO (1 μM) and/or the PKC inhibitor BIMI (3 μM) as indicated below (*n* = 8−19). Statistical significance was determined using ANOVA multi-comparison test when ***p ≤ 0.001, and ns, not statistically significant.

To date, TRPM5 has been considered to be a calcium-activated ion channel^2,43^. This mechanism is achieved in physiological settings by activating Gq/GPCR, followed by calcium release from the stores. Our results suggest that PKC phosphorylation is required for channel-evoked currents, mainly at relevant physiological membrane potentials (Vm = approximately −60 mV). To determine the involvement of each component (calcium and PKC phosphorylation), we recorded the evoked currents of HEK293T cells co-expressing WT or mutated mTRPM5 (S129A) with the Gq/DREADD receptor using nominally free calcium conditions (no calcium or chelator in the recording pipette). We used this experimental setup to analyze the temporal contribution of PKC phosphorylation to the physiological process of TRPM5 activation. Under nominally free conditions, no baseline current was observed in the WT or mutated receptor (Fig. 5a, b). However, upon CNO application, a robust current was evoked in the WT receptor compared with the mutated receptor (Fig. 5a, b). Of note, the CNO-evoked current of the mTRPM5 under nominally free conditions was similar to that observed with a saturated fixed calcium concentration (compare Fig. 5a and Fig. 1b).

As mentioned above, TRPM5 is activated at negative potentials under physiological conditions. Therefore, we examined the CNO-evoked current under nominally free conditions at V_m_ = −80 mV (Fig. 5c). No current was observed at the S129A mutated receptor, highlighting the necessity of PKC phosphorylation for TRPM5 activation. Thus, our results suggest that calcium release and PKC phosphorylation are required for TRPM5 activation under physiological conditions.

Finally, to further analyze the role of PKC phosphorylation of TRPM5 in the activation process, we examined the effect of BIM I under nominally free conditions at both positive and negative potentials (Fig. 5d, e). Our results showed that at both potentials, inhibition of PKC (i.e., BIM I application) or the PKC phosphorylation mutation (i.e., S129A) significantly minimized the evoked calcium response. Therefore, PKC phosphorylation is essential for the activation mechanism of TRPM5.

## Discussion

**Fig. 6:**
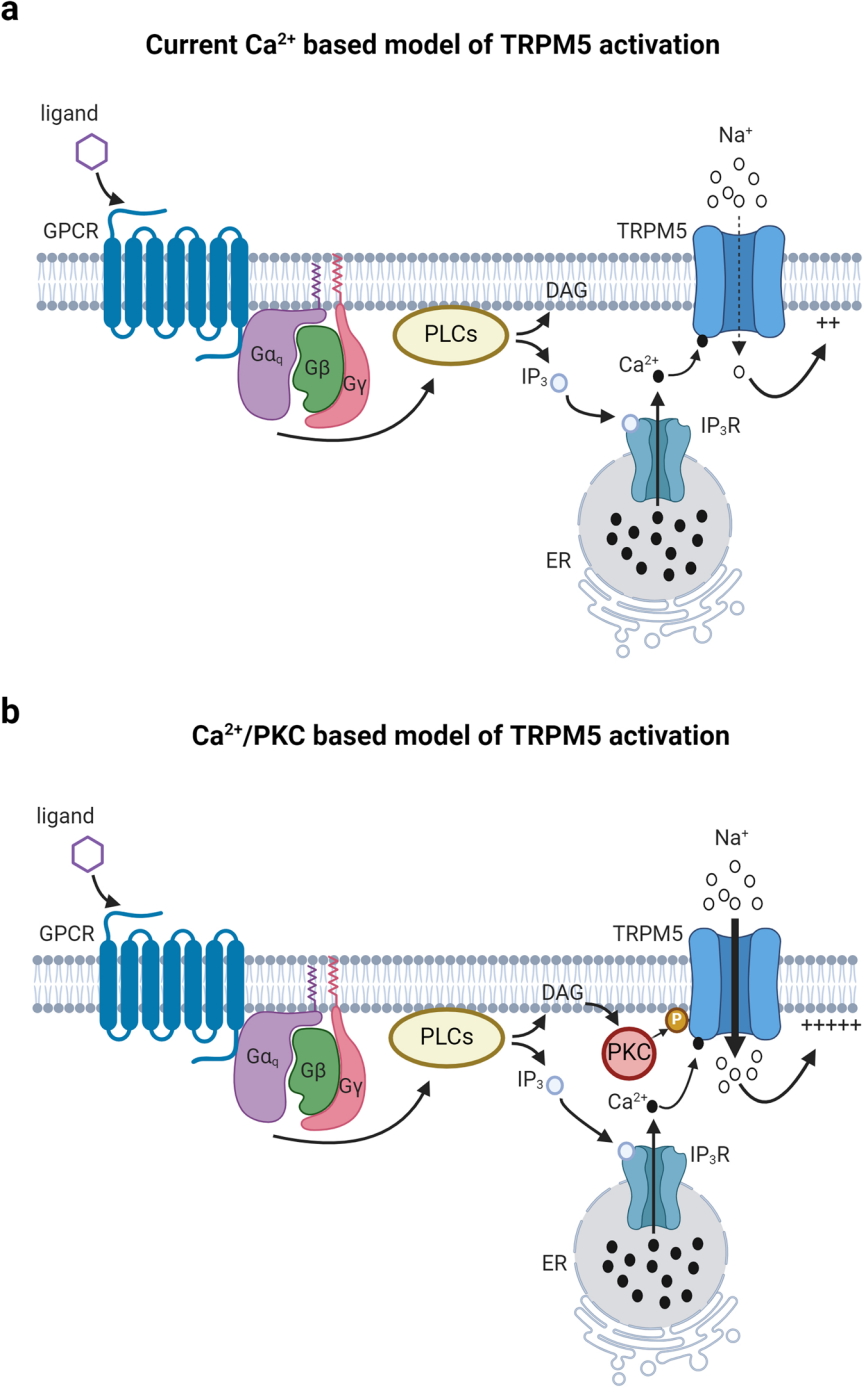
Schematic models of the calcium-dependent TRPM5 activation and the calcium- and PKC-dependent TRPM5 activation*.* **a** TRPM5 activation requires the following cascade: ligand (purple hexamer) binding to the G protein-coupled receptor (GPCR) leads to the dissociation of the heterotrimeric G proteins. Consequently, the Gαq subunit of the G protein activates PLCs, triggering the breakdown of PIP2 into DAG and IP3. IP3, in turn, activates IP3 receptors (IP3R), which causes the release of Ca^2+^ from the endoplasmic reticulum (ER). Next, calcium ions bind and activate TRPM5. We and others demonstrate that the solely calcium-dependent activation resulted in a subdued inward current (dashed arrow). **b** Our results indicate that simultaneously with the intracellular calcium release, DAG activates the protein kinase C (PKC), which phosphorylates the TRPM5 channel. This phosphorylation combines with the bound calcium, leads to a robust inward current (bold arrow). Created with BioRender.com.

Our study provides insight into the requirements of both intracellular calcium release and PKC phosphorylation for TRPM5 activation and modulation by the Gq/GPCR pathway. The activation mechanism(s) of TRPM5 has been controversial^44^, with different mechanisms suggested, including the central issue of intracellular calcium (Fig. 6a) or other second messengers as the sole activator of TRPM5. Our findings indicated that the positive modulation of TRPM5 is not merely dependent on intracellular calcium levels (Fig. 1). By isolating different components of the Gq/GPCR pathway, we showed that TRPM5 activation depends on PKC phosphorylation (Fig. 2).

By further investigating the role of PKC phosphorylation in TRPM5, we demonstrated that multiple phosphorylation sites regulate the Gq/TRPM5 modulation axis, with the serine at position 129 playing a substantial role in this positive modulation (Table 1). Thus, neutralization of serine 129 abolished TRPM5 modulation through a direct mechanism unrelated to the calcium sensitivity of the channel, as observed in the dose-response analysis of the mutated channel form (Fig. 3e, f). Moreover, the substitution of serine 129 to aspartate (S129D) resulted in higher baseline current at both positive and negative potentials, mimicking the PKC-dependent phosphorylated form of TRPM5 (Fig. 4).

Also, our results revealed that both calcium release and PKC phosphorylation are crucial for the positive modulation of TRPM5 by the Gq pathway (Fig. 6b). Notably, the effect of PKC phosphorylation at negative voltages was significantly more pronounced than that at positive voltages (Fig. 5d, e), which is particularly important under physiological conditions. However, lower currents (compared to WT TRPM5) were observed at positive voltages under nominally free calcium conditions (Fig. 5). This may be attributed to the voltage-dependent modulation of TRPM5 currents at high voltages.

Phosphorylation by protein kinase C (PKC) is an essential aspect of TRPM channel regulation, encompassing TRPM4 and TRPM5^45^, and has functional implications in various cellular contexts. Previous studies have demonstrated that PKC-mediated phosphorylation of TRPM4 modulates channel activity, leading to changes in sodium ion flux. This regulatory mechanism is critical for maintaining proper membrane potential and controlling electrical signaling in tissues, such as the heart and neurons^46^. Similarly, PKC phosphorylation of TRPM5 has been implicated in taste transduction, particularly in the bitter and umami taste receptors^47^. Furthermore, PKC activity in the TRPM4 and TRPM5 channels is crucial for facilitating GLP-1-induced insulin secretion, offering valuable insights into the physiological processes related to glucose metabolism and the functioning of pancreatic beta cells^45^. These findings highlight the significant role of PKC in modulating the activity and functional properties of TRPM5 under physiological conditions, thereby introducing it as a potential therapeutic target in related fields.

A notable aspect of our study is that the lack of specific agonizts targeting TRPM5 poses a challenge for the development of therapeutic interventions. Therefore, discovering novel approaches or strategies to modulate TRPM5 activity is crucial for addressing related health conditions^48^. Furthermore, it opens avenues for exploring alternative mechanisms that can indirectly regulate TRPM5 or modulate downstream signaling pathways. Thus, understanding the exact signaling pathway of TRPM5 activation would enhance the discovery of potential drug developments for TRPM5 and hold great significance in pharmacology and therapeutics.

Our results indicate that calcium release and PKC phosphorylation, specifically at position 129, are essential for activating TRPM5 via the Gq pathway (Fig. 6b). These findings provide novel insights into the signaling pathways that govern TRPM5 activation. Thus, it provides implications for understanding its physiological roles in sensory and other biological processes, and offers potential therapeutic targets for diseases or conditions involving TRPM5 dysregulation^49^. In conclusion, this study enhances our understanding of the intricate signaling pathways that regulate TRPM5 and highlights the therapeutic potential of targeting this signaling pathway.

### Supplementary information

Supplementary information

Description of Supplementary Materials

Supplementary Data

Reporting Summary

## Data Availability

The source data for the graphs in this study are provided in Supplementary Data. Figure 6 was created with BioRender.com. All other supporting data generated and/or analyzed during this study are available from the corresponding author upon reasonable request.
