# Supplementary material for: TRPM5 activation depends on a synergistic effect of calcium and PKC phosphorylation
Source: Commun Biol. 2024 Mar 27;7:369. doi: 10.1038/s42003-024-06054-3 (PMC10973328; doi:10.1038/s42003-024-06054-3)
Supplement: Supplementary file 1 — Supplementary information [file 42003_2024_6054_MOESM1_ESM.pdf]

Supplementary Information

TRPM5 activation depends on a synergistic effect of calcium and PKC phosphorylation

Alaa Nmarneh & Avi Priel

The Institute for Drug Research, School of Pharmacy, Faculty of Medicine, The Hebrew University of Jerusalem, Ein Karem, Jerusalem, 9112102, Israel

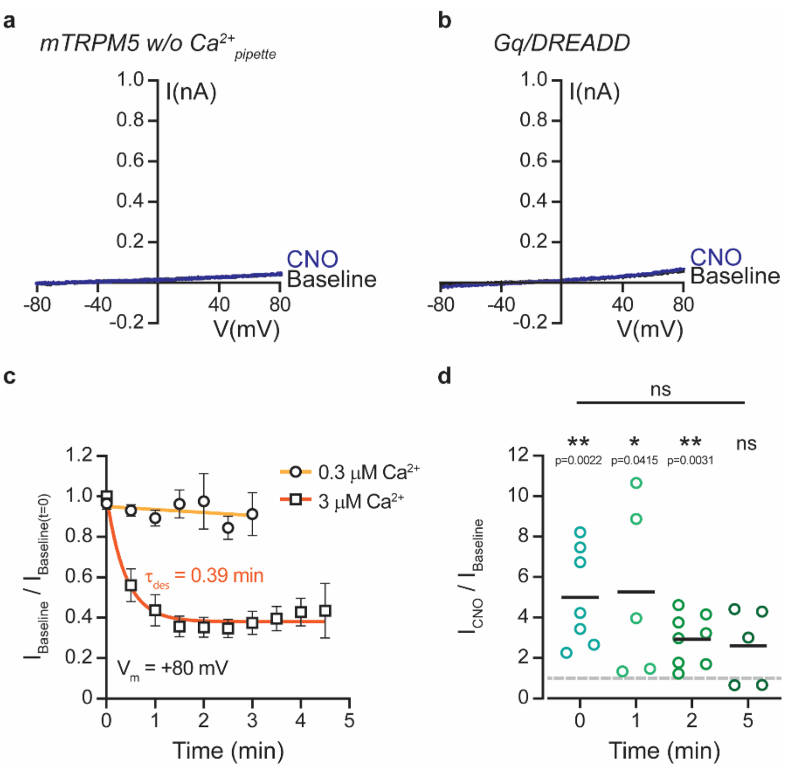

Suppl. Figure 1. The GqPCR positive modulation of TRPM5 currents is not only Ca^{2+}-dependent.

a & b. Representative current-voltage relationship traces in HEK293T cells transiently expressing the wt mTRPM5 (a) or the Gq/DREADD (b) (n = 5-7). Currents were recorded with 3 µM Ca^{2+} in the recording pipette (b) or without (a) before (Baseline; black trace) and after CNO application (CNO; 1 µM; blue line).

c. Exponential decay plot representing the normalized baseline current evoked by two different pipette calcium concentrations (0.3 µM; circles, and 3 µM; squares)) in HEK293T cells expressing the wt mTRPM5. Currents were recorded for 3-5 minutes immediately after establishing the whole-cell configuration (t=0) at +80 mV. Each of the obtained set data points was fitted with one exponential phase fit (light and dark orange lines). The desensitization time constant (τ_{des}) was determined only when substantial desensitization was obtained. Each point represents the average (±SEM) response of n=5 cells. ***d.*** Mean/Scatter dot plot representing the CNO (1 µM)-current amplification with Ca^{2+} (3 µM) in the recording pipette of HEK293T cells transiently co-expressing mTRPM5 with Gq/DREADD (n = 5-8). Currents were recorded within different time scales after whole-cell establishment at +80 mV. Statistical significance for each data set before (dash grey line) and after the CNO application was determined using paired t-test when ns, not statistically significant. Statistical significance between the whole data sets was determined by ANOVA multi-comparison test when ns, not statistically significant. Note that CNO-evoked significant current augmentation even after two minutes of exposure to intracellular Ca^{2+}.

Suppl. Figure 2. The Gq/GPCR activation increases the TRPM5 calcium-evoked currents even at high non-physiological calcium concentrations.

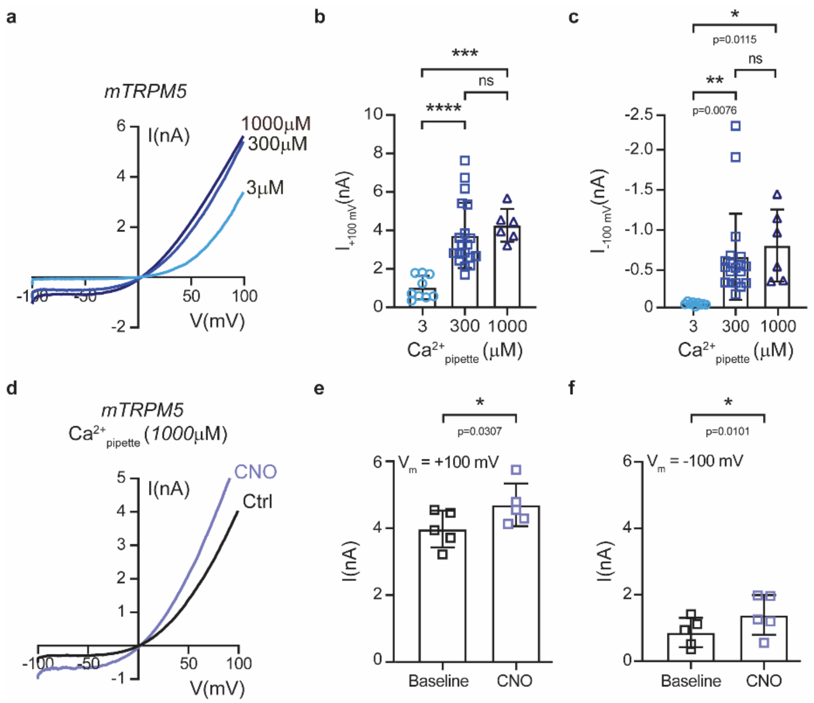

a. Representative current-voltage relationship traces in HEK293T cells transiently expressed mTRPM5. Baseline currents were recorded with various calcium concentrations (as indicated) in the recording pipette. Currents were recorded using the whole-cell configuration (1 s^{-1} voltage ramps between −100 and +100 mV) (n= 6-19). ***b & c.*** Mean/Scatter dot plot representing the baseline current at (+100 mV) (b) and (-100mV) (c) of various calcium concentrations 3, 300, and 1000 µM in the recording pipette (as shown in A) (n = 6-19). Statistical significance was determined using the ANOVA multi-comparison test when****p≤0.0001, ***p≤0.001, and ns, not statistically significant. ***d.*** Representative current-voltage relationship traces in HEK293T cells transiently expressing the mTRPM5 with Gq/DREADD. Currents were recorded with 1000 µM Ca^{2+} in the recording pipette before (Baseline; black trace) and following CNO application (CNO; 1 µM; blue line). Currents were recorded using the whole-cell configuration (1 s^{-1} voltage ramps between −100 and +100 mV) (n= 5). ***e & f.*** Mean/Scatter dot plot representing the current of 1000 µM Ca^{2+} in the recording pipette at +100 mV (e) and -100 mV (f) of HEK293T cells transiently expressing mTRPM5 (WT) with Gq/DREADD before (Baseline), and following CNO application (CNO;1 µM) (n = 5). Statistical significance was determined using paired t-test.

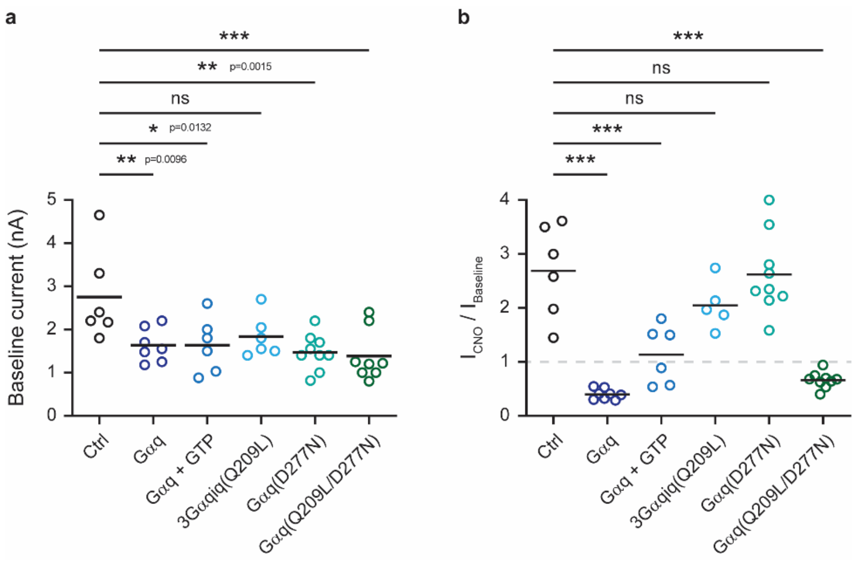

Suppl. Figure 3. The Gα_q subunit is necessary but not sufficient for the GqPCR-modulation of the TRPM5 current. Mean/scatter dot plot (n = 5-9) representing the TRPM5 response to 3 μM Ca^{2+} (***a***) or the fold increase response evoked by 1 μM CNO (***b***) with 3 μM Ca^{2+} in the recording pipette. HEK 293T Cells were transiently co-expressing mTRPM5, Gq/DREADD, and the indicated Gαq subunit; native WT Gαq, 3Gαqiq (Q209L) chimera: in this chimera, the PLCβ-binding region on the Gα_q domain is replaced by the corresponding part of the Gα_{i2} subunit and included the Q209L mutation. Thus, this chimera is a constitutively active Gα_q subunit that disabled to couple PLCβ. The Gα_q dominant negative mutants; Gα_q(D277N), which strengthens the Gα-Gβγ protein interface and reduces the response caused by the wild-type Gα_q, and Gα_q(Q209L/D277N) mutant, which loses the ability to activate downstream effectors via mimicking the nucleotide-free Gα form. Statistical significance was determined using ANOVA multi-comparison test when ***p≤0.001, and ns, not statistically significant.

Suppl. Table 1. Primers.
The oligos sequence was used for the generation of the different constructs.

Oligo name	Oligo sequence
EGFP-F(HINDIII) - Fusion	AAGCTGAAGCTTACCATGGTGAGCAAG
mTRPM5-B (ECOR I)-Fusion	AGTGAATTCTCCATTTCAGGTGTCAGAGGG
EGFP-M5-F- Fusion	GACGAGCTGTACAAGATGCAAACAACCCAGAGC
EGFP-M5-B- Fusion	GACGAGCTGTACAAGATGCAAACAACCCAGAGC
Gαq pC3.1+_F (CHIMERA)	ACCGAGAATATCCGCTTTG
Gαq pC3.1+_B(CHIMERA)	CTCATTGTCTGACTCCAC
Gαi2 pc3.1+_F(CHIMERA)	TCGTGGAGTCAGACAATGAGAACCGCATGCATGAGAGC
Gαi2 pc3.1+_ B(CHIMERA)	ACAAAGCGGATATTCTCGGTGGTGGCGCACGTGAAGTG
EGFP-M5(S33A)-F	CTTCGGAGGGGCTGGGAAGAAGC
EGFP-M5(S33A)-B	TTGATCTCTCCCCTGCATAGGATG
EGFP-M5(S98A)-F	AGCAGCTCAGGCCACAGGTGCCTGG
EGFP-M5(S98A)-B	TTCACCAGCCCCTTGCGC
EGFP-M5(S127A)-F	CTCTCTGGCTGCCACATCCACCAAG
EGFP-M5(S127A)-B	TGATCACGTACAGCTTGTC
EGFP-M5(S129A)-F	GGCTAGCACAGCCACCAAGATCC
EGFP-M5(S129A)-B	AGAGAGTGATCACGTACAGCTTG
EGFP-M5(T130V)-F	TAGCACATCCGTCAAGATCCGTGTAG
EGFP-M5(T130V)-B	GCCAGAGAGTGATCACGT
EGFP-M5(T534A)-F	TGAGATGGCCGCATACTTCTGGG
EGFP-M5(T534A)-B	TAACGATTCTGCAGCACAG
EGFP-M5(T569A)-F	GGTGGCCCGCGCCATGCGTGAGG
EGFP-M5(T569A)-B	TCTGCCTCTTTCTCCAGGTGGGACATTTCC
EGFP-M5(T633A)-F	AGCATTCCTGGCCAAGATCTGGTG
EGFP-M5(T633A)-B	TGCACACCGTCATGGGCA
EGFP-M5(T721A)-F	CTTCCTGCTCGCACGGTGGAGGA
EGFP-M5(T721A)-B	GCAGCTTGTGGGCCTAGATC
EGFP-M5(T822A)-F	TGTGGGAGTCGCCTGTAGAATGG
EGFP-M5(T822A)-B	ATGAACAGGAAGATGGCCAC
EGFP-M5(T845A)-F	CATGGTGTTCGCACTTCGGCTCA
EGFP-M5(T845A)-B	AAGTCAATGGCCAGAACGG
EGFP-M5(T1055A)-F	TACCTGGGAAGCGGTTCAAAAGG
EGFP-M5(T1055A)-B	ATGATCTTCTGGTCCAAGG
EGFP-M5(T1064A)-F	CTTCCTGAGTGCCATGGAGAAAC
EGFP-M5(T1064A)-B	TTCTCCTTTTGAACCGTTTC
EGFP-M5(S1106A)-F	GTGTCTGGAAGCACAGGCCAACT
EGFP-M5(S1106A)-B	TTGATCCTCTTTTCTTGCTCTCTCAG
EGFP-M5(S1142A)-F	TCAGCCAGCCGCTGCTAGAGACA
EGFP-M5(S1142A)-B	CTCCTGCAACCACAGTTCTG
